# Methods to drive systems biology forward

**DOI:** 10.15252/msb.20178155

**Published:** 2017-12-21

**Authors:** Maria Polychronidou, Thomas Lemberger

**Affiliations:** ^1^ EMBO Heidelberg Germany

**Keywords:** Methods & Resources

## Abstract

The development of new methodologies has driven the expansion of systems biology over the past decades. Technological breakthroughs in sequencing, in quantitative proteomics, in single‐cell measurements, to name only a few, have each opened up whole new fields of research. To highlight the importance of new experimental and computational methodologies in enabling novel biological discoveries, we are pleased to announce the introduction of a new Methods section in *Molecular Systems Biology* (http://msb.embopress.org/authorguide#methodsguide).

Since its launch, the journal has maintained a constant interest for papers with strong methodological components. A few recent examples include the i3C method for analyzing chromatin conformation without chemical cross‐linking (Brant *et al*, 2016), a deep learning classifier for high‐content imaging data (Kraus *et al*, 2017), and an RNA‐seq‐based approach for synthetic circuit characterization and debugging (Gorochowski *et al*, 2017). After discussions with authors and readers and consultation with our Editorial Advisory Board, we concluded that a dedicated Methods section would provide a more visible home to studies with a primary focus on methodological developments in all flavors of systems and synthetic biology. In line with the scope of the journal, Method articles published in *Molecular Systems Biology* should report technologies that are likely to be broadly adopted by the scientific community and have a clear potential to reveal new biology. Even if new fundamental discoveries are not necessarily expected, new methods should be applied to a concrete biological question.

Our new Methods section will present both experimental and computational methods that put forward novel concepts, open new areas of research, or allow the formulation of new biological questions. We will also publish original approaches that show significantly improved performance over existing methodologies or allow analyses at scales or at levels of accuracy that were not possible thus far. When possible, measures of performance, validation of predictions, and systematic benchmarking with independent datasets should be reported. To ensure reproducibility and wide dissemination, it is particularly important that Methods are rigorously documented. Ease of implementation, precise description of protocols, and availability of the relevant reagents, software components, and datasets are further important criteria since they all facilitate the broad adoption of a new method across different labs.

We are proud to launch our Methods section with a string of papers reporting exciting new technologies (Lawson *et al*, 2017; Schmierer *et al*, 2017 and Weile *et al*, 2017). Weile *et al* (2017) systematically map functional missense variation in human genes to identify disease variants. They present an innovative approach based on a new deep mutational scanning strategy combined with machine learning that produces complete functional maps for full‐length proteins. The studies by Lawson *et al* (2017) and Schmierer *et al* describe methodologies for characterizing genotype–phenotype relationships. In the first study, Lawson *et al* (2017) combine single‐molecule live‐cell microscopy phenotyping with *in situ* genotyping by sequential fluorescent hybridization of barcoded plasmids. Their method enables for the first time the application of sensitive time‐lapse imaging to the analysis of large genetic libraries and thus allows mapping genetic diversity to dynamic molecular phenotypes. Schmierer *et al*, 2017, address the important issue of reproducibility and robustness in genome‐scale CRISPR‐based functional screens. They show that massively parallel lineage tracing through the inclusion of random sequence labels provides an elegant method to improve the accuracy, precision, and statistical power of large‐scale functional screens.

We are confident that the addition of this new Methods section to the content of *Molecular Systems Biology* will appeal to a broad range of researchers, and we enthusiastically invite the community to submit high‐quality method papers reporting innovative methodological advances that will drive new discoveries and bring the field forward.

## References

[msb178155-bib-0001] Brant L , Georgomanolis T , Nikolic M , Brackley CA , Kolovos P , van Ijcken W , Grosveld FG , Marenduzzo D , Papantonis A (2016) Exploiting native forces to capture chromosome conformation in mammalian cell nuclei. Mol Syst Biol 12: 891 27940490 10.15252/msb.20167311 PMC5199122

[msb178155-bib-0002] Gorochowski TE , Espah Borujeni A , Park Y , Nielsen AA , Zhang J , Der BS , Gordon DB , Voigt CA (2017) Genetic circuit characterization and debugging using RNA‐seq. Mol Syst Biol 13: 952 29122925 10.15252/msb.20167461 PMC5731345

[msb178155-bib-0003] Kraus OZ , Grys BT , Ba J , Chong Y , Frey BJ , Boone C , Andrews BJ (2017) Automated analysis of high‐content microscopy data with deep learning. Mol Syst Biol 13: 924 28420678 10.15252/msb.20177551 PMC5408780

[msb178155-bib-0004] Lawson MJ , Camsund D , Larsson J , Baltekin Ö , Fange D , Elf J (2017) *In situ* genotyping of a pooled strain library after characterizing complex phenotypes. Mol Syst Biol 13: 947 29042431 10.15252/msb.20177951 PMC5658705

[msb178155-bib-0005] Schmierer B , Botla SK , Zhang J , Turunen M , Kivioja T , Taipale J (2017) CRISPR/Cas9 screening using unique molecular identifiers. Mol Syst Biol 13: 945 28993443 10.15252/msb.20177834 PMC5658704

[msb178155-bib-0006] Weile J , Sun S , Cote AG , Knapp J , Verby M , Mellor J , Wu Y , Pons C , Wong C , van Lieshout N , Yang F , Tasan M , Tan G , Yang S , Fowler DM , Nussbaum R , Bloom JD , Vidal M , Hill DE , Aloy P *et al* (2017) A framework for exhaustively mapping functional missense variants. Mol Syst Biol 13: 957 10.15252/msb.20177908 PMC5740498 29269382

